# Nurses’ perceptions of patient safety culture: a mixed-methods study

**DOI:** 10.1186/s12913-020-05441-w

**Published:** 2020-06-26

**Authors:** Nina Granel, Josep Maria Manresa-Domínguez, Carolina Eva Watson, Rebeca Gómez-Ibáñez, Maria Dolors Bernabeu-Tamayo

**Affiliations:** Nursing Department, Medicine Faculty, Universitat Autònoma de Barcelona, Av. de Can Domènech, 737, 08193 Bellaterra, Barcelona, Spain

**Keywords:** Nursing care management, Organizational culture, Patient safety, Safety management

## Abstract

**Background:**

There are relatively few qualitative studies concerning patient safety culture.

**Methods:**

We aimed to explore patient safety culture as perceived by the nursing staff in two public hospitals in Catalonia, Spain. A mixed-methods design was employed using a questionnaire, in-depth interviews, and non-participant observations.

**Results:**

Sixty-two percent of the nursing staff rated patient safety as “Acceptable” but was not higher because of work pressure and lack of resources as perceived by staff. “Teamwork within units” had the highest rate of positive responses, and “Staffing” had the lowest rate. Emergency units showed more negative results than the other two units.

**Conclusions:**

Safety incidents are not always reported due to fear of punishment, reflecting a lack of positive safety culture. It is necessary to design and implement strategies that promote a positive culture to avoid punitive responses and apply and evaluate these changes.

## Background

In European countries with advanced healthcare systems, adverse events due to healthcare provided occur in 8 to 12% of hospitalizations [1]. Adverse events have a significant impact on patient morbidity and mortality [2]. In Spain, 9–12% of patients treated in hospitals, including in emergency departments, suffer an adverse event. Around 50–70% of these adverse events could be avoided by applying demonstrated safety practices [3].

Patient safety requires an organizational and multidisciplinary approach, in which nursing is fundamental. Nurses can help prevent multiple avoidable adverse events, such as medication errors, pressure ulcers, lack of information, falls, and nosocomial infections, among others [4]. To improve patient safety, institutions need to promote, create, and maintain a positive patient safety culture [5, 6]. An institution’s patient safety culture is the sum of individual and group values, attitudes, perceptions, competencies, and behavior patterns that determine the style of, competence in, and commitment to safety management in the institution [7, 8]. Institutions with a positive safety culture are characterized by communication based on mutual trust, perceptions of the importance of safety, and reliance on effective preventive actions. Staff and institutions recognize mistakes, learn from them, and improve. Promoting a safety culture in health systems is an international challenge [7].

The most widely used instrument for assessing safety culture is the *Hospital Survey on Patient Safety Culture* (HSOPSC) by the United States (US) Agency for Healthcare Research and Quality (AHRQ) [8]. This questionnaire assesses the perception of patient safety culture among hospital staff, including an institution’s values, beliefs, and standards; event reporting; communication; leadership; and management [9]. This instrument has been validated in several European Union (EU) countries: Finland, Iceland, Norway, Switzerland, and the Netherlands [10]; Belgium [11]; Croatia [12]; France [13]; Italy [14]; the United Kingdom [15]; Portugal [16]; Spain [17]; and Sweden [18].

Quantitative studies on patient safety culture, such as the ones cited above, are abundant in the literature; however, qualitative or mixed-methods studies in this area are limited. In 2014, a thematic literature review was published in which only one qualitative study concerning safety culture was identified [19]. Aside from a mixed-methods Iranian study [20] and an Ethiopian cross-sectional study [21], no other qualitative studies have been identified. Experts in patient safety culture have suggested that mixed-methods approaches, using interviews or focus groups, can help researchers understand safety culture in greater depth [19].

The aim of this study was to explore patient safety culture as perceived by the nursing staff in the Surgical, Internal Medicine, and Emergency departments of two public hospitals in Catalonia, Spain. The specific objectives were to: 1) Describe the strengths and weaknesses of the perceived safety culture in the specific services provided, 2) Determine the perceived global patient safety grade, and 3) Identify the number of incidents reported during the last year.

## Methods

This study examined the patient safety culture in two hospitals located in Catalonia through a convergent parallel design mixed-methods study following the guidelines recommended by Creswell [22]. This approach was not for the purpose of comparing results, which is a common reason for conducting a parallel design; instead, the researchers included transformed data in the convergent analysis process. This integrative design allowed us to maintain fidelity and separation among one sequence of mixed-methods processes which resulted in quantitative findings and one qualitative sequence which provided phenomenological context around the data, both having the same priority and remaining separate during the analysis. The findings from these two methodological sequences were then unified and triangulated, which originated a set of data to be interpreted, and an overall interpretation was performed providing a greater understanding of the patient safety culture at the participating hospitals units [22].

### Sample and setting

The study population consisted of nursing staff from the Internal Medicine, General Surgery, and Emergency units of two public hospitals from diverse regions of Catalonia. The hospitals had certain characteristics and dimensions in common (same nurse/patient ratio and overall patient numbers). The total beds per unit were between 28 and 30, having an average ratio of 1 nurse/10 beds in the Medicine and Surgery units. In the Emergency units each nurse was in charge of approximately 5 patients. The occupancy rate was 95–100% for all the units. The units selected accounted for the highest percentage of overall hospital nursing staff employed.

### Data collection

Permit from the hospitals’ research boards was obtained in March 2017, and data were collected from April to May 2017. The main researcher presented the project to the units’ supervisors, and they distributed the Spanish validated version of the HSOPSC questionnaire [17] to the nursing staff who had been working in the hospital for a minimum of 1 year, along with a letter explaining the project and covering informed consent. No exclusion criteria were established. Once implemented, questionnaires and consent forms were deposited securely and anonymously in a box. To increase the response rate, weekly reminders were made by the supervisors and the principal investigator to each work shift during the data collection period. Finally, questionnaires and consent forms were returned to the investigators.

The HSOPSC contains 42 items classified into 12 dimensions (Tables 1 and 3). Nine items use a 5-point Likert scale to indicate the level of agreement (“*strongly disagree*” to “*strongly agree*”), and the other three items use a 5-point Likert to indicate frequency (“*neve*r” to “*always*”). The instrument also measures sociodemographic data, as well as the patient safety grade and number of incidents reported in the previous year.

**Table 1:** Comparisons of patient safety culture dimensions between hospitals

Safety culture dimensions	Hospital 1 (*n* = 63)	Hospital 2 (*n* = 46)	p
1. Frequency of event reporting	43.92% (41.8)	42.75% (40.2)	.885
2. Overall perceptions of safety	31.0% (28.0)	25.5% (24.4)	.295
3. Supervisor/manager expectations and actions promoting safety	50.4% (33.1)	45.7% (34.2)	.468
4. Organizational learning—continuous improvement	41.8% (33.3)	43.5% (35.0)	.800
5. Teamwork within hospital units	70.2% (34.4)	63.6% (34.0)	.319
6. Communication openness	55.6% (34.9)	37.0% (30.8)	.005
7. Feedback and communication about error	27.0% (31.0)	32.6% (27.6)	.322
8. Non-punitive response to error	39.7% (30.4)	35.5% (31.7)	.489
9. Staffing	16.3% (16.8)	11.4% (13.6)	.112
10. Hospital management support for patient safety	13.2% (23.6)	24.6% (33.2)	.055
11. Teamwork across hospital units	45.6% (31.6)	48.4% (39.8)	.679
12. Handoffs and transitions	54.4% (30.9)	59.8% (30.0)	.363

The main outcome variable was positive response rate, which was calculated as follows: the number of positive answers to each question in a dimension divided by the total number of answers to all questions in the dimension. Negatively-worded items were reverse-coded before calculation. A department was considered to show a good safety culture in each dimension or an area of strength when a dimension’s percentage of positive responses was ≥75%; a low safety culture needing improvement was ≤50%.

**Fig. 1:**
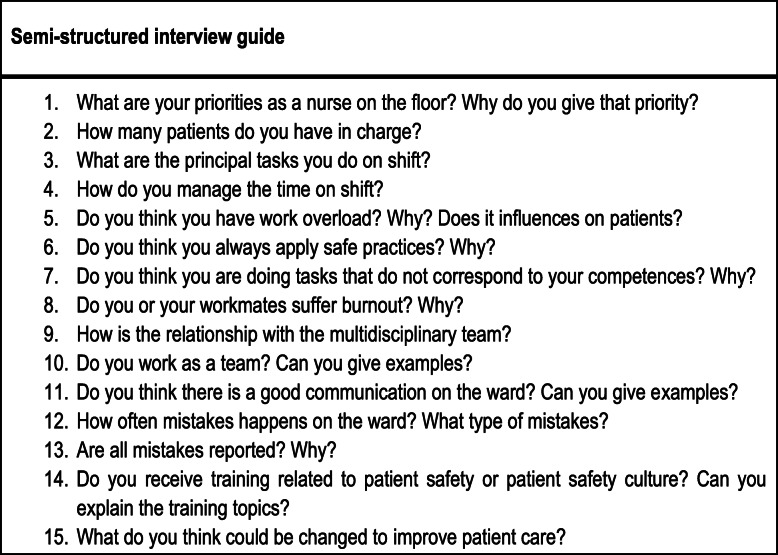
Topic guide for the semi-structured interviews

To collect qualitative data, nine semi-structured in-depth interviews were conducted, along with a total of 42 h of non-participant observation of nurses in the three units during their seven-hour shifts were carried out by spending 2 days in each unit. The principal researcher wore the institutional uniform and was introduced to the staff on shift and answered research questions to avoid the Howthorne effect of observation. The routines, performance, and actions of the nurses at their workplace and the teamwork environment were observed; field notes were taken during the observations. Participants for the interviews were selected purposively using critical case sampling approach [22], in which nurses who were likely to provide the most information were selected. The inclusion criterion was a minimum of 3 years of work experience in the unit (“efficient” or “expert” according to Benner’s theory) [23], with the objective of guaranteeing a deep knowledge of the services provided and the conditions in which they were provided. The main researcher during the non-participant observation selected and invited nurses to set voluntarily the time to conduct the interviews outside the work area in a meeting room facilitated by the hospital. Interviews with three nurses from each participant unit were recorded until data saturation was reached [22] following an interview guide (Fig. 1). All the interviews were conducted in Spanish, with each interview lasting 30–50 min. The transcripts of the interviews were reviewed by the participants to ensure that their views were accurately reflected. The audio-recorded interviews were transcribed verbatim and translated into English by the principal author.

### Data analyses

SPSS 23.0 for Windows (Armonk, NY: IBM Corp.) was used to conduct descriptive statistical quantitative analyses. The significance level used in the bilateral contrasts was *p* ≤ .05. The categorical variables were summarized with their absolute and relative frequencies, and the continuous ones with their means and standard deviations. Pearson’s chi-squared test was used to test proportions, and variance analysis with Tukey’s post-hoc correction or Dunnett’s T3 was used to test means, depending on the variance homogeneity.

Participants’ responses to the interviews and observation field notes were coded in terms of the 12 categories established by the AHRQ that compose the patient safety culture. Atlas.ti version 7.5.12 for Windows was used. Quotations were coded by collection technique (OBS: non-participant observation and INT: in-depth interview), individual participants’ numbers, and work units (MED: medicine, SURG: surgery, and EMERG: emergency).

## Results

One-hundred and fifty-four questionnaires were distributed to all nurses in the participating units in both hospitals, and 109 were completed (response rate = 70.7%). Ninety-three percent of the participants were women. Overall, 75 nurses (68.8%) had been working for more than 11 years. Internal Medicine units had a higher difference in hours worked per week compared to Surgical and Emergency units (Table 2). Interviews provided evidence across all dimensions, but only the dimensions considered to be strengths or those requiring improvement were subjected to further analysis, which provided the most relevant results.

**Table 2:** Patient safety grade, incident report and other work data between hospitals and units

	Hospital 1 (*n* = 63)	Hospital 2 (*n* = 46)	p	Internal Medicine (*n* = 26)	General Surgery (*n* = 34)	Emergency (*n* = 49)	p	Total (*n* = 109)
Patient safety grade			.325				.010	
Excellent/good	10 (16.1%)	12 (26.1%)		9 (34.6%)	4 (12.1%)	9 (18.4%)		22 (20.4%)
Acceptable	39 (62.9%)	28 (60.9%)		14 (53.8%)	27 (81.8%)	26 (53.1%)		67 (62.0%)
Poor	13 (21.0%)	6 (13.0%)		3 (11.5%)	2 (6.1%)	14 (28.6%)		19 (17.6%)
Number of incidents reported			.118^†^				.049^†^	
None	50 (79.4%)	29 (63.0%)		14 (53.8%)	26 (76.5%)	39 (79.6%)		79 (72.5%)
1 to 2 incidents	12 (19.0%)	12 (26.1%)		6 (23.1%)	8 (23.5%)	10 (20.4%)		24 (22.0%)
3 to 5 incidents	1 (1.6%)	4 (8.7%)		5 (19.2%)	0 (0.0%)	0 (0.0%)		5 (4.6%)
6 to 10 incidents	0 (0.0%)	1 (2.2%)		1 (3.8%)	0 (0.0%)	0 (0.0%)		1 (0.9%)
Working hours per week			.039				.013	
More than 40 h	11 (17.5%)	16 (34.8%)		12 (46.2%)	5 (14.7%)	10 (20.4%)		27 (24.8%)
Career in the hospital			.938				.185	
1 to 5 years	12 (19.1%)	6 (13.0%)		3 (11.5%)	7 (20.6%)	8 (16.3%)		18 (16.5%)
6 to 10 years	9 (14.3%)	7 (15.2%)		6 (23.1%)	6 (17.6%)	4 (8.2%)		16 (14.7%)
11 to 15 years	19 (30.2%)	14 (30.4%)		8 (30.8%)	9 (26.5%)	16 (32.7%)		33 (30.3%)
16 to 20 years	8 (12.7%)	6 (13.0%)		1 (3.8%)	2 (5.9%)	11 (22.4%)		14 (12.8%)
More than 21 years	15 (23.8%)	13 (28.3%)		8 (30.8%)	10 (29.4%)	10 (20.4%)		28 (25.7%)

^†^*p*-value of the contrast between “none” versus “1 or more incidents”

The majority of the participants from which qualitative data were collected were women (8 out of 9), and had more than 10 years of clinical experience, and between 5 and 17 years of experience in their units.

### Patient safety could be improved

Patient safety grade is shown in Table 2. Per the qualitative data, most interviewees felt that patient safety could be improved and mentioned that lack of resources and work pressure put the patients at risk.

*The nurse is giving medication to the patient; suddenly, she gets a phone call to explain the condition of a patient, who has to move from one unit to another. The nurse complains that she has heavy interruptions. OBS 3 MED.*

*[You go home thinking] thank goodness, I’ve finished, because I would have caused a disaster. Often you leave with that feeling, or the feeling that you have forgotten things because you don’t have enough time! INT 6 SURG.*

Emergency staff in both hospitals perceived a worse patient safety grade than Internal Medicine and General Surgery staff (*p* = .010). Qualitative data were in accordance with these results.

*For example, I have an allergy reaction in the corridor without a monitor. Also, a 94-year-old lady in the corridor who, at any time, can become disorientated and fall from the stretcher. Then, what do you want me to say? There are a lot of things going wrong every day related to patient safety. INT 5 EMER.*

### Incidents were not always reported

Incident reporting data are shown in Table 2.

During the observations, other incidents were also detected that were not reported by the staff:

*The nurse from the morning shift calls another unit to explain the conditions of a patient to be admitted, she explains … that the medications prescribed at night time haven’t been validated by the night shift nurse on the computer and, therefore, she’s not aware if it’s been given to the patient. They assume it’s been given without the night nurse’s confirmation and don’t report the incident. OBS 1 EMERG.*

During the interviews, most nurses associated “incidents” only with medication errors or issues and verbalized that, sometimes, they discussed them with the doctor. However, neither doctors nor nurses notified it in writing through the formal reporting process.

*I think that the majority get notified, but all? I don’t think so. If you realize, you tell the doctor; but, it doesn’t go any further*. *INT 4 MED.*

*Once, a nurse made a mistake; she administrated three 1 ml morphine ampoules when she had to administer 0.3 ml. She told the doctor, but they did not report it. INT 6 SURG.*

In Internal Medicine, a slightly higher percentage of incident reporting was observed compared to the other departments (Table 2).

### Teamwork within units was the most valued dimension

Dimension 5, “teamwork within units”, showed high percentages of positive answers in the two hospitals (70.2 and 63.6%, respectively). Staff supported each other, treated each other with respect, worked together, and usually helped their colleagues.

*The medical office is next to the nursing office, and that helps a lot. It contributes to good communication. The communication with healthcare assistants is good; we delegate work to them and … there is no problem. INT 6 SURG.*

*One nurse on shift asks other nurses and healthcare assistants if they have finished the patients’ hygiene rounds and if they needed help. OBS 3, 4 MED, and 2, 6 SURG.*

However, difficulties existed concerning teamwork between nurses and doctors.

*I think communication is good between doctors and nurses, but it also depends with whom you communicate better, you know, who to address to. INT 6 SURG.*

By unit, dimension 5 showed an especially strong percentage in Surgical units (83.1%); no other dimensions were considered strengths by unit (Table 3).

**Table 3:** Comparisons of patient safety culture dimensions between units

Safety culture dimensions	Internal Medicine (*n* = 26)	General Surgery (*n* = 34)	Emergency (*n* = 49)	p
1. Frequency of event reporting	53.9% (41.2)	42.2% (41.2)	38.8% (40.4)	.312
2. Overall perceptions of safety	26.9% (22.3)	36.0% (26.2)	24.5% (28.2)	.139
3. Supervisor/manager expectations and actions promoting safety	50.0% (32.4)	48.5% (33.1)	47.5% (35.1)	.953
4. Organizational learning—continuous improvement	51.3% (30.2)	52.0% (35.0)	31.3% (32.2)	.007^†^
5. Teamwork within hospital units	74.0% (27.8)	83.1% (25.2)	53.1% (37.4)	< .001^‡^
6. Communication openness	52.6% (34.2)	52.9% (33.9)	41.5% (34.3)	.235
7. Feedback and communication about error	35.9% (28.2)	32.4% (33.3)	23.8% (27.2)	.190
8. Non-punitive response to error	32.1% (30.5)	44.1% (34.5)	36.7% (28.2)	.308
9. Staffing	12.5% (14.6)	16.9% (18.2)	13.3% (14.5)	.479
10. Hospital management support for patient safety	26.9% (32.7)	15.7% (23.5)	15.0% (28.9)	.191
11. Teamwork across hospital units	55.8% (31.9)	44.1% (33.2)	43.9% (35.2)	.304
12. Handoffs and transitions	64.4% (28.4)	58.1% (29.3)	51.5% (32.0)	.210

^†^Tukey post-hoc contrast: differences between Emergency and Internal Medicine (*p* = .035) and between Emergency and General Surgery (*p* = .015)

^‡^Tukey post-hoc contrast: differences between Emergency and Internal Medicine (*p* = .021) and between Emergency and General Surgery (*p* < .001)

### Staffing rates and work pressure needed improvement

Dimension 9, “staffing,” showed the lowest percentages (16.3 and 11.4%, respectively). Staff unanimously thought that there were not enough personnel and that, sometimes, patients’ care suffered because of the lack of personnel. This data were reinforced by the interviews and observations.

*Always, there is always pressure, if not the doctors, the relatives … the workload … it is a lot. If you want to do things right, it is a lot. INT 3 MED.*

*“I am fed up,” “I am very tired,” “always the same.” From these comments, you detect that staff are tired. INT 4 MED.*

*There is work pressure in some shifts with less staff. The workload is the same, and supervisors can see that you can’t manage it, and you ask for extra staff and they don’t arrive, perhaps because nobody’s available or because it’s not possible at that moment. INT 2 SURG.*

The following attitudes were detected through observation: excessive concern related to lack of time for tasks, irritability, tension, and tiredness.

### The safety culture of the emergency units slightly differed from other hospital units

Emergency units showed lower positive response rates on 10 out of the 12 dimensions, with significant differences on dimensions 4 “organization learning—continuous improvement” (*p* = .007) and 5 “teamwork within units” (*p* < .001). Nurses in the Emergency units detected a lack of resources to apply and evaluate preventive measures to avoid mistakes, and appropriate measures were not applied to prevent mistakes from reoccurring. They did not receive enough training in these areas and emphasized a lack of resources to allow improvements.

*We have 20 h per year for training, which is very little. We have done a two-hour training on patient safety. It focused on errors, incidents … I left with the feeling that it was good, but the problem is lack of resources. INT 1 EMERG.*

In Emergency units, staff had their own routine: nurses had patients assigned and worked independently. Although they worked as a team when necessary, they sometimes did not manifest mutual support and respect.

*The staff have a lack of care sometimes. There are those who are more involved and others who are not. This is a service with a lot of staff and movement; it is easier to leave your position. It is not like a ward. INT 1 EMERG.*

The lack of teamwork with doctors was confirmed repeatedly, supporting the significant difference in dimension 5.

*I miss the communication we had before the system was digitized because we used to learn a lot, and it allowed teamwork with the doctors. INT 5 EMERG.*

*When everything was digitized, communication levels decreased. For example, with medication changes, there are doctors who communicate them, but there are others who don’t; you have to go behind them to see the changes and the plan they have for the patient. INT 1 EMERG.*

### The Catalan hospitals studied had globally similar safety culture

There was no significant difference between hospitals except on dimension 6 “communication openness”; the staff at Hospital one spoke more freely and questioned their supervisors more (Table 1).

However, it was not possible to verify this through the qualitative data, owing to the diversity of responses and some discomfort among the interviewees in speaking about errors. They did not always answer questions clearly.

*Some staff keep it quiet, and others report that things happen. INT 2 SURG.*

*We discuss everything with our supervisor without being afraid because there is enough trust. INT 3 MED.*

The interviewees stated that this lack of communication openness was related to perceived culpability and the fear of punishment.

*Well, I guess it’ll be for fear of reprisals or for … the person above you … you know, he can blame you in some way or, if it’s not that, what other reason can there be? If nothing happens, no one finds out. INT 3 MED.*

## Discussion

The mixed-methods research provided an overview of the state of patient safety culture among staff in two Catalan hospitals and elucidated a deeper understanding of the perceptions of nurses in these hospitals and, by extension, in Spanish hospitals generally. Most participants had more than 11 years of nursing experience, which according to previous studies, indicates that they have good knowledge of the institutions and their safety culture [24, 25].

Elevated concerns about insufficient time to complete tasks, irritability, tension, and tiredness are signs of stress [26], which, in turn, can be associated with work pressure while on shift and, for some nurses, working overtime. Differences in staff ratios and work pressure influence on institution’s safety culture [21]. Thus, nurses did not rate safety culture higher than “acceptable” as a result of work pressure and perceived lack of resources.

It has been previously described in the literature that certain risky behaviors can become norms under work pressure [27]. Furthermore, the lack of open communication in notifying the hospital through formal reporting of avoidable adverse events because of fear of punishment and perceived culpability [28], along with confirmed misreporting detected through the observations and with acknowledgment in interviews that there are unreported events, qualifies the low number of reported incidents.

Four studies have been conducted in Spain using the HSOPSC [6, 17, 29, 30]. All indicated that “teamwork within units” was the dimension with the most positive results and “staffing” was the lowest, matching the current results. This suggests that the strengths and weaknesses of patient safety culture are similar throughout Spanish hospitals, and they have not changed over time despite the fact that Spain has several different regional healthcare systems.

A 2011 study showed low percentages of positive responses in 30 Emergency departments in Spain [29]. A comparable result was found in the present study and in a previous paper [17], where distinct departments were compared. Of note, percentages were lower in Emergency departments in both studies than in other departments.

In most other European studies, the dimension “teamwork within units” has been considered a strength, except in some eastern European countries [13, 16, 18, 31]. Regarding dimensions needing improvement, the results differ between Spain and other EU countries: “manager support for patient safety” and “staffing” were rated the lowest of all the dimensions in Sweden [32]; however, “none—punitive response to error” and “manager support for patient safety” were rated the lowest, just below “staffing,” in Portugal [16]. One French study indicated “none—punitive response to error” as the lowest [13], while an Italian study reported “teamwork among hospital units” just as low as “staffing.” [14]

The characteristics of organizations with a positive safety culture [33]—such as trust in the effectiveness of preventative measures, communication based on trust, and team perceptions about the importance of safety—are areas in which to improve in the institutions studied. In no case had the evaluation of staff or institutions been attempted. The main purpose of previous work has been to understand survey results in order to propose future improvement strategies.

Our findings can assist nurse managers to identify the weaknesses and strengths in relation to hospital safety issues based on nursing perceptions and behaviors. Nurse managers could develop and implement specific strategies to improve patient safety, including providing education related to patient safety culture to the staff members (including managers). Managers could also promote a positive culture through communication by encouraging staff to notify incidents and avoiding punitive responses.

### Limitations

Strategies to ensure rigor such as building a research team, elaborating guides for data collection, defining and obtaining adequate participation and reaching data saturation were achieved. A limitation of this study is its focus on nurses, who comprised around 40% of healthcare staff in the studied hospitals. Future mixed-methods studies must be conducted to obtain evidence from other professionals and form a complete view of the hospitals’ patient safety culture in Catalan Hospitals.

## Conclusions

Teamwork within units is considered to be a valuable quality in these institutions globally; nevertheless, staff numbers and work conditions could be improved. Specifically, incidents are not always reported by nursing staff for fear of punishment, pointing to a lack of positive safety culture. Therefore, we assert the need to work on positive safety culture in the participating hospitals through specific strategies of improvement. The quantitative results from the HSOPSC mostly match those of other studies carried out in Spain and the qualitative data analyzed; however, there are differences when comparing results with those from hospitals in other European countries.

## Data Availability

The datasets used and/or analysed during the current study are available from the corresponding author on reasonable request.

## Abbreviations

AHRQ: Agency for Healthcare Research and Quality
EMERG: Emergency
EU: European Union
HSOPSC: Hospital Survey on Patient Safety Culture
IBM: International Business Machines
INT: In-depth interview
MED: Medicine
NY: New York
OBS: Non-participant observation
SPSS: Statistical Package For Social Sciences
SURG: Surgery
US: United States
